# Supplementary material for: Controlled release of basic fibroblast growth factor from a peptide biomaterial for bone regeneration
Source: R Soc Open Sci. 2020 Apr 1;7(4):191830. doi: 10.1098/rsos.191830 (PMC7211882; doi:10.1098/rsos.191830)
Supplement: Supplements revising [file rsos191830supp1.docx]

Supplements

1. **Mechanical test of the materials**

| Table 1. mechanical results of nHA/PA66/D-RADA16 and nHA/PA66. | | | |
| --- | --- | --- | --- |
|  | average macropore size(um) | porosity（%） | compressive strength（Mpa） |
| nHA/PA66 | 633.21±111.37 | 82.67±4.41 | 3.21±0.98 |
| nHA/PA66/D-RADA16 | 621.76±109.11 | 79.31±8.33 | 3.31±1.09 |
| NS (p > 0.05) | | | |

**2. Rheology test**

Rheology test on 1^o^ stainless steel cone-controlled rheometer with a diameter of 20 mm (Thermo Fisher Science, Waltham, MA, USA) Samples were dissolved in PBS (pH 7.4) at a concentration of 20.0 mg/ml. The peptide solution was diluted with PBS (pH 7.4) to 10mg/ml, 5mg/ml and 2.5mg/ml respectively and stored at 4C in a refrigerator overnight, and 150–200 ul of samples were used for analysis at 25C.


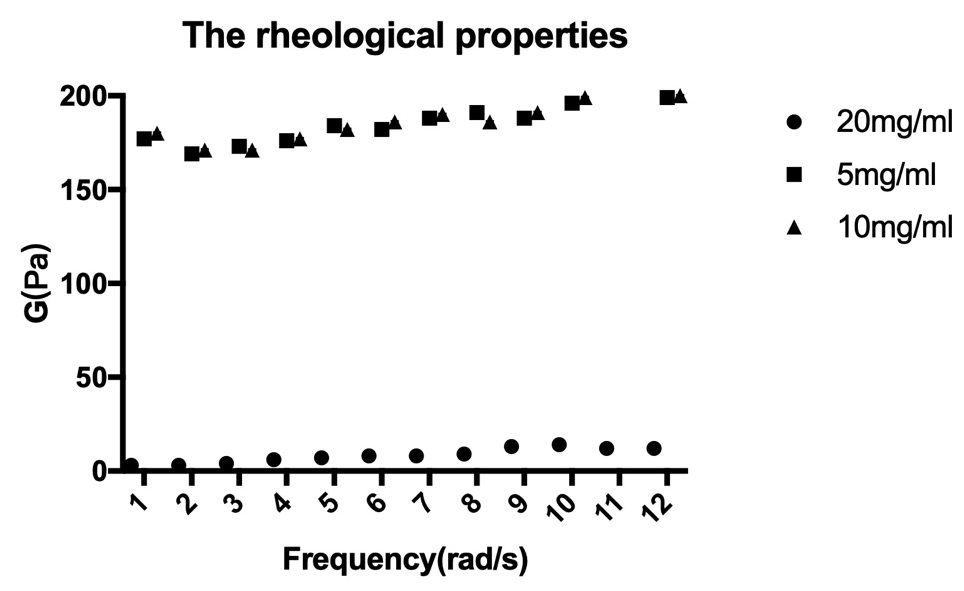


1. **Peptides**

Schematic illustration for a molecular model of D-RADA16 peptide in a progressive self-assembly process: I) A single D-RADA16 peptide molecule, II) a few peptides in solution before self-assembly, III) self-assembling peptides form β-sheet structure, IV) peptides further form nanofibers.


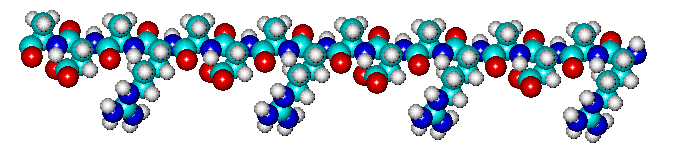


Molecular models of pair of chiral peptides. (D-RADA16）All peptides are modeled in the extended (N–>C). Color code: hydrogen = white, carbon = cyan, oxygen = red and nitrogen = blue.

**4.** **abbreviations**

nano-hydroxyapatite/polyamide 66 (nHA/PA66);

transmission electron microscopy (TEM);

scanning electron microscopy (SEM);

bone mesenchymal stem cells (BMSCs);

Confocal laser microscopy (CLS);

alkaline phosphatase (ALP);

basic fibroblast growth factor (bFGF);

Cell Counting Kit-8 (CCK-8)

5-ethynyl-2-deoxyuridine (EdU)

Sprague Dawley rats (SD rats)
